# Association of Medicaid Expansion With Medicaid Enrollment and Health Care Use Among Older Adults With Low Income and Chronic Condition Limitations

**DOI:** 10.1001/jamahealthforum.2022.1373

**Published:** 2022-06-03

**Authors:** Melissa McInerney, Grace McCormack, Jennifer M. Mellor, Lindsay M. Sabik

**Affiliations:** 1 Department of Economics, Tufts University, Medford, Massachusetts; 2 National Bureau of Economic Research, Cambridge, Massachusetts; 3 Harvard Kennedy School, Harvard University, Cambridge, Massachusetts; 4 Department of Economics, William & Mary, Williamsburg, Virginia; 5 Schroeder Center for Health Policy, William & Mary, Williamsburg, Virginia; 6 Department of Health Policy and Management, University of Pittsburgh School of Public Health, Pittsburgh, Pennsylvania

## Abstract

**Importance:**

Medicaid is an important source of supplemental coverage for older Medicare beneficiaries with low income. Research has shown that Medicaid expansion under the Patient Protection and Affordable Care Act (ACA) was associated with increased Medicaid coverage for previously eligible older adults with low income, but there has been little research on whether their health care use increased or whether such changes differed by beneficiaries’ health status.

**Objective:**

To assess whether the ACA Medicaid expansion to working-age adults was associated with increased Medicaid enrollment and health care use among older adults with low income with and without chronic condition limitations.

**Design, Setting, and Participants:**

This cross-sectional study used data from the National Health Interview Survey from 2010 to 2017 for adults 65 years or older with low income (≤100% of the federal poverty level). Data were analyzed from November 2020 to March 2022.

**Exposure:**

Residence in a state with Medicaid expansion for working-age adults.

**Main Outcomes and Measures:**

The main outcomes were Medicaid coverage and health care use, measured by physician office visits and inpatient hospital care. Survey weights were used in calculating descriptive statistics and regression estimates. In multivariate analysis, difference-in-differences models were used to compare changes in outcomes over time between respondents in Medicaid expansion states and respondents in nonexpansion states.

**Results:**

Of 21 859 adults included in the study, 7153 had chronic condition limitations (4983 [70.1%] female; mean [SD] age, 76.0 [0.1] years) and 14 706 did not have chronic condition limitations (9609 [66.3%] female; mean [SD] age, 74.85 [0.08] years). Of those with chronic condition limitations, 2707 (36.7%) were enrolled in Medicaid, 2816 (39.4%) had an office visit in the past 2 weeks, and 2152 (30.7%) used inpatient hospital care in the past year. Medicaid expansion was associated with differential increases in the likelihood of having Medicaid (4.92 percentage points; 95% CI, 0.25-9.60 percentage points; *P* = .04) and having an office visit in the past 2 weeks (5.31 percentage points; 95% CI, 0.10-10.51 percentage points; *P* = .046) compared with nonexpansion. There were no differential changes between expansion and nonexpansion states in receipt of inpatient hospital care (−0.62 percentage points; 95% CI, −5.39 to 4.14 percentage points; *P* = .79). Among adults without chronic condition limitations, 3159 (19.8%) were enrolled in Medicaid, and no differential changes between expansion and nonexpansion states in Medicaid enrollment (−0.24 percentage points; 95% CI, −3.06 to 2.57 percentage points; *P* = .86) or health care use were found.

**Conclusions and Relevance:**

In this cross-sectional study, ACA Medicaid expansion for working-age adults was associated with increased Medicaid enrollment and outpatient health care use among older adults with low income and chronic condition limitations who were dually eligible for Medicare and Medicaid.

## Key Points

**Question:**

Was the expansion of Medicaid to working-age adults under the Patient Protection and Affordable Care Act (ACA) associated with changes in Medicaid enrollment and health care use among older adults with low income and chronic condition limitations?

**Findings:**

In this cross-sectional study of 7153 US adults 65 years or older with low income, ACA Medicaid expansion was associated with significant increases in the likelihood of Medicaid enrollment and outpatient health care use among those with chronic condition limitations. No associations were found between ACA Medicaid expansion and Medicaid enrollment and health care use among those without such limitations.

**Meaning:**

In this study, expansion of Medicaid to working-age adults was associated with increased Medicaid enrollment and outpatient health care use among older adults with low income and chronic condition limitations who were enrolled in Medicare.

## Teaser

This cross-sectional study uses data from the National Health Interview Survey to assess the association of Medicaid expansion to working-age adults with Medicaid enrollment and health care use among older adults with low income and chronic condition limitations.

## Introduction

Medicaid expansion under the Patient Protection and Affordable Care Act (ACA) was associated with substantial benefits for the population eligible for Medicaid after expansion (adults aged 19-64 years with low income). In expansion states, the likelihood of being uninsured decreased significantly among newly eligible working-age adults,^1^ with increased health care access and use. Prior studies showed that Medicaid expansion was associated with increased use of primary and preventive care,^2,3,4^ outpatient care,^5^ and prescription drug use^6^ among working-age adults, with mixed associations with inpatient hospital care.^7^ Prior work suggests that coverage changes resulting from Medicaid expansion were concentrated among working-age adults with poor health.^8,9,10^ Similarly, prior studies showed that Medicaid expansion was associated with larger improvements in health, reductions in cost-related barriers to care, and increases in outpatient and prescription drug use among working-age adults with poor health.^6,9,10,11,12,13,14,15,16^ These findings may have been attributable to adults with poor health being most likely to benefit from increased coverage.

In addition, Medicaid expansion was associated with indirect benefits, including increased enrollment among groups previously eligible for, but not enrolled in, Medicaid.^17,18,19,20,21,22^ After expansion to working-age adults, Medicaid enrollment increased by more than 4% among adults 65 years or older with low income.^23^ These enrollment increases among the previously eligible (called “welcome mat” effects) have been attributed to increased awareness of program benefits and reduced enrollment costs.^20^ For older adults, Medicaid enrollment provides valuable supplemental coverage of Medicare premiums and cost-sharing and services that traditional Medicare does not cover (eg, home and community-based services, dental, and vision). However, prior estimates suggest that only approximately half of older adults who meet eligibility requirements enroll in Medicaid.^24,25^

We examined whether Medicaid expansion was associated with increased health care use among older adults. To our knowledge, the only prior study to examine the ACA Medicaid expansions and health care use among older adults found that primary care use by Medicare enrollees did not increase in the first 2 years after expansion.^26^ We examined multiple measures of health care use up to 4 years after expansion. Because of evidence that the consequences of Medicaid expansion for working-age adults differed by their health status, we examined whether changes in coverage and health care use were concentrated among older adults with low income and poor health. Because most older adults have been told they have a chronic condition,^27^ we stratified the sample based on the presence of a limitation from a chronic condition.

## Methods

### Data

This cross-sectional study used data from 2010 to 2017 from the National Health Interview Survey (NHIS), an annual cross-sectional in-person survey of the civilian noninstitutionalized population conducted by the National Center for Health Statistics (NCHS).^28^ The NHIS collects information on health insurance; health care use; and self-reported health, income, and other demographic characteristics. We accessed the restricted-use NHIS with state of residence identifiers, available at Federal Statistical Research Data Centers. Analysis of restricted data through the NCHS at the Federal Statistical Research Data Centers was approved by the NCHS Research Ethics Review Board. This study was deemed exempt under category 4 of the Tufts University institutional review board protocol 1705069; a waiver of informed consent was not required because of the exemption. This study followed the Strengthening the Reporting of Observational Studies in Epidemiology (STROBE) reporting guideline.

### Study Sample

We included survey respondents 65 years or older who were enrolled in Medicare and had income at or below 100% of the federal poverty level (eFigure 1 in the Supplement). This allowed us to identify respondents who were likely eligible for full or partial Medicaid based on their income. We identified respondents who met our inclusion criteria using respondent-reported income, and for respondents not reporting income, NHIS-provided imputed values of income were used. Because 5 imputed values were provided, we applied multiple imputation methods and report the sample size from the first imputation.

We stratified the sample according to whether or not respondents reported at least 1 limitation from a chronic condition at the time of the survey. In the NHIS, 78% of older adults with low income had ever been told they had a chronic condition, but only 33% were currently experiencing a limitation from a chronic condition.

NHIS respondents are asked whether they are “limited in any way in any activities because of physical, mental, or emotional problems?” Enumerators give additional questions to identify the underlying cause(s), including acute (eg, fracture) and chronic conditions. We selected the following conditions also used by the NCHS to measure the prevalence of chronic conditions: arthritis or rheumatism, heart problems, stroke problems, hypertension or high blood pressure, diabetes, lung or breathing problems, or cancer.^29^

### Outcomes

Primary outcomes included binary indicators of whether the respondent had Medicaid coverage at the time of completing the survey and used medical care 10 or more times in the past year as well as binary and continuous measures of use of physician office visits in the past 2 weeks and inpatient hospital care in the past year. Secondary outcomes included whether the respondent had to delay or forgo medical care owing to cost in the past year, had problems paying medical bills in the past year, and had fair or poor self-reported health at the time of the survey.

### Explanatory Variable of Interest and Covariates

The main independent variable was an indicator for whether the respondent lived in an expansion state after it expanded Medicaid under the ACA. Covariates included measures of respondent age and its square, educational level (less than high school degree, high school degree or General Educational Development Test, some college, or college degree or more), sex, and race and ethnicity (Hispanic, non-Hispanic Black, non-Hispanic White, or other [non-Hispanic Alaska Native, American Indian, Asian Indian, Chinese, Filipino, Other Asian, multiracial with no primary race selected, or primary race not releasable]). Respondent race and ethnicity were self-reported or imputed by NCHS. We included state indicators to account for unobserved permanent differences across states and year indicators to control for temporal shocks common to all states.

### Statistical Analysis

Data were analyzed from November 2020 to March 2022. In descriptive analyses, we compared observable traits for respondents with and without chronic condition limitations and preexpansion means of outcomes by expansion state residence. In multivariate analysis, we estimated difference-in-differences models comparing changes in outcomes over time between respondents in Medicaid expansion states and respondents in nonexpansion states with the following formula:

Y*_ist_* = *β EXPANSION_s_* × *POST_t_* + *γ_s_* + *τ_t_* + *X_i_* + *ϵ_ist_*,

where *EXPANSION* indicates individual *i* lived in a state that expanded Medicaid between 2014 and 2017 and *POST* indicates the survey took place after that state’s expansion. β is the difference-in-differences estimate of the association of Medicaid expansion and the outcome, *Y_ist_*. Of 32 states that expanded Medicaid before the end of 2017, 27 expanded in 2014 and 5 in subsequent years. The vectors *γ_s_* and *τ_t_* represent state and year fixed effects, respectively. Given that 39% of individuals 65 years or older did not report exact income and were assigned 5 NHIS-imputed income values, we used multiple imputation to generate estimates and SEs.^30^ We used survey weights in calculating descriptive statistics and regression estimates and clustered regression SEs by state. To support our identifying assumption, we tested whether pre–expansion period trends in outcomes were parallel in the 2 groups of states using an event study specification.

We conducted various supplemental analyses, as described in the eAppendix in the Supplement. We tested whether Medicaid expansion was associated with respondents’ likelihood of reporting a chronic condition limitation. We conducted multiple robustness checks of our main results by (1) including potentially endogenous covariates, such as marital status, family size, income, employment status, number of chronic conditions, number of limitations in activities of daily living, and the state unemployment rate; (2) changing sample inclusion criteria to include individuals with income up to 150% of the federal poverty level, income below their state-specific eligibility limit for full Medicaid, and those not enrolled in Medicare; and (3) excluding respondents residing in states that expanded Medicaid before 2014, already provided Medicaid or similar coverage to adults with low income between 2010 and 2013,^2^ or expanded Medicaid in the middle of a year. Finally, we estimated our main difference-in-differences model for 2 additional measures of outpatient care: receipt of advice or test results via telephone and receipt of home care visits from a health professional. All analyses were conducted using Stata\IC, version 16.1 (StataCorp LLC), and 2-sided *P* < .05 was considered statistically significant.

## Results

### Study Sample

Of 21 859 adults included in the study, 7153 had chronic condition limitations (4983 [70.1%] female; mean [SD] age, 76.0 [0.1] years) and 14 706 did not have chronic condition limitations (9609 [66.3%] female; mean [SD] age, 74.85 [0.08] years). A total of 4280 respondents (17.0%) identified as Hispanic, 4154 (16.3%) as non-Hispanic Black, 11 049 (58.2%) as non-Hispanic White, and 2376 (8.5%) as other race or ethnicity.

Table 1 shows data for demographic characteristics among older adults with low income by presence or absence of a chronic condition limitation. Respondents with chronic condition limitations were a mean of 1.2 years (95% CI, 0.9-1.4; *P* < .001) older, 9.5 percentage points (95% CI, 7.8-11.0 percentage points; *P* < .001) more likely to have less than a high school degree, and 4.4 percentage points (95% CI, 3.1-5.7 percentage points; *P* < .001) more likely to be non-Hispanic Black.

**Table 1.** Demographic Characteristics, Medicaid Coverage, and Health Care Use Among Adults 65 Years or Older With Low Income^a^

Characteristic	Respondents^b^		Difference (95% CI)^d^	*P* value
	With chronic condition limitations (n = 7153)^c^	Without chronic condition limitations (n = 14 706)		
Age, mean (SD), y	76.01 (0.11)	74.85 (0.08)	1.17 (0.89 to 1.42)	<.001
Educational level				
Less than high school	3413 (45.7)	5626 (36.2)	9.5 (7.8 to 11.0)	<.001
High school degree or GED	2064 (30.0)	4922 (34.7)	−4.7 (−6.2 to −3.2)	<.001
Some college	1126 (15.9)	2639 (18.6)	−2.7 (−3.9 to −1.3)	<.001
College or more	550 (8.4)	1519 (10.5)	−2.1 (−3.1 to −1.1)	<.001
Sex				
Female	4983 (70.1)	9609 (66.3)	3.8 (2.2 to 5.3)	<.001
Male	2170 (29.9)	5097 (33.7)	−3.8 (−5.4 to −2.1)	<.001
Race and ethnicity				
Hispanic	1259 (15.3)	3021 (17.8)	−2.5 (−4.1 to −0.8)	.003
Non-Hispanic Black	1643 (19.3)	2511 (14.9)	4.4 (3.1 to 5.7)	<.001
Non-Hispanic White	3615 (58.3)	7434 (58.2)	0.1 (−1.9 to 2.1)	.93
Other^e^	636 (7.1)	1740 (9.2)	−2.0 (−3.1 to −1.0)	<.001
Resides in expansion state	4308 (60.0)	9014 (62.0)	−2.0 (−4.1 to −0.03)	.046
Had Medicaid coverage at time of survey	2707 (36.7)	3159 (19.8)	16.9 (15.1 to 18.5)	<.001
Received medical care in person ≥10 times in past year	2800 (40.1)	2331 (16.1)	24.0 (22.4 to 25.5)	<.001
Had an office visit in past 2 wk	2816 (39.4)	3667 (24.8)	14.6 (12.9 to 16.3)	<.001
Office visits in past 2 wk, mean (SD), No.	0.68 (0.02)	0.36 (0.01)	0.33 (0.28 to 0.37)	<.001
Office visits in past 2 wk among those with a visit, mean (SD), No.	1.74 (0.04)	1.44 (0.02)	0.29 (0.21 to 0.38)	<.001
Had an overnight hospital stay in past year	2152 (30.7)	1956 (13.8)	16.9 (15.3 to 18.5)	<.001
Hospital stays in past year, mean (SD), No.	0.68 (0.05)	0.21 (0.01)	0.47 (0.37 to 0.57)	<.001
Hospital stays in past year among those with a stay, mean (SD), No.	2.24 (0.15)	1.54 (0.04)	0.70 (0.38 to 1.01)	<.001
Had to delay or forgo care owing to cost at least once in past year	687 (9.4)	794 (5.4)	4.0 (3.1 to 4.9)	<.001
Had problems paying medical bills in past year	1457 (22.8)	1772 (13.7)	9.1 (7.5 to 10.7)	<.001
Self-reported health was fair or poor at time of survey	4795 (66.3)	4026 (26.9)	39.4 (37.6 to 40.9)	<.001

Abbreviation: GED, General Educational Development Test.

^a^ Data are from respondents to the National Health Interview Survey from 2010 to 2017. Low income was defined as income at or below 100% of the federal poverty level.

^b^ Data are presented as unweighted number (weighted percentage) unless otherwise indicated. Unweighted sample size was reported from 1 imputation wave. Weighted percentages and means were estimated using multiple imputation of 5 different samples that were defined based on 5 different imputed incomes.

^c^ Those with chronic conditions had a limitation from any 1 of the following conditions: arthritis or rheumatism, heart problems, stroke problems, hypertension or high blood pressure, diabetes, lung or breathing problems, or cancer.

^d^ Data are presented as percentage points for categorical data and counts for continuous data.

^e^ Other included non-Hispanic Alaska Native, American Indian, Asian Indian, Chinese, Filipino, Other Asian, multiracial with no primary race selected, and the primary race not releasable.

Respondents with chronic condition limitations were 2 percentage points (95% CI, −4.1 to −0.03 percentage points; *P* = .046) less likely to live in an expansion state. A total of 2707 respondents with chronic condition limitations (36.7%) were enrolled in Medicaid, compared with 3159 respondents without chronic condition limitations (19.8%) (difference, 16.9 percentage points; 95% CI, 15.1-18.5 percentage points; *P* < .001). Respondents with chronic condition limitations used more health care and were more likely to report barriers to care. For example, 2816 respondents with chronic condition limitations (39.4%) had an office visit in the past 2 weeks compared with 3667 (24.8%) of those without a limitation (difference, 14.6 percentage points; 95% CI, 12.9-16.3 percentage points; *P* < .001) and 2152 respondents with chronic condition limitations (30.7%) had an overnight hospital stay in the past year compared with 1956 respondents without a limitation (13.8%) (difference, 16.9 percentage points; 95% CI, 15.3-18.5 percentage points; *P* < .001).

Table 2 shows data on outcomes for individuals surveyed before the first year of Medicaid expansion by the presence of a chronic condition limitation and their state’s expansion status. In each chronic condition limitation group, most outcomes showed no statistically significant differences by expansion status.

**Table 2.** Medicaid Coverage, Health Care Use and Access, and Financial and Health Outcomes^a^

Characteristic	Respondents with chronic condition limitations^b^					Respondents without chronic condition limitations				
	Expansion states (n = 2155)^c^	Nonexpansion states (n = 1459)^c^	Difference (95% CI)^d^	*P* value	Expansion states (n = 4531)^c^		Nonexpansion states (n = 2758)^c^	Difference (95% CI)^d^	*P* value
Had Medicaid coverage at time of survey	799 (35.4)	578 (35.5)	−0.1 (−4.5 to 4.3)	.96	917 (17.8)		607 (18.4)	−0.5 (−2.9 to 1.9)	.66
Received medical care in person ≥10 times in past year	882 (41.4)	581 (40.8)	0.6 (−3.3 to 4.5)	.76	754 (17.1)		441 (16.0)	1.1 (−1.0 to 3.2)	.29
Had an office visit in past 2 wk	823 (38.8)	585 (39.8)	−1.0 (−5.1 to 3.1)	.63	1078 (23.8)		694 (25.3)	−1.4 (−3.8 to 0.9)	.23
Office visits in past 2 wk, mean (SD), No.	0.65 (0.03)	0.67 (0.04)	−0.02 (−0.12 to 0.08)	.69	0.33 (0.01)		0.37 (0.02)	−0.04 (−0.08 to 0.01)	.13
Office visits in past 2 wk among those with a visit, mean (SD), No.	1.69 (0.05)	1.69 (0.05)	0.002 (−0.15 to 0.16)	.97	1.40 (0.03)		1.46 (0.05)	−0.06 (−0.17 to 0.05)	.29
Had an overnight hospital stay in past year	685 (33.4)	442 (30.0)	3.4 (−0.3 to 7.1)	.07	584 (13.4)		398 (14.9)	−1.6 (−3.4 to 0.3)	.10
Hospital stays in past year, mean (SD), No.	0.71 (0.04)	0.57 (0.04)	0.14 (0.02 to 0.25)	.02	0.20 (0.01)		0.23 (0.02)	−0.03 (−0.07 to 0.01)	.13
Hospital stays in past year among those with a stay, mean (SD), No.	2.15 (0.09)	1.93 (0.08)	0.22 (−0.02 to 0.45)	.07	1.52 (0.05)		1.55 (0.07)	−0.04 (−0.21 to 0.13)	.67
Had to delay or forgo care owing to cost at least once in past year	189 (8.4)	149 (9.7)	−1.2 (−3.3 to 0.8)	.24	237 (5.5)		166 (6.1)	−0.5 (−1.9 to 0.8)	.43
Had problems paying medical bills in past year	378 (21.3)	323 (27.5)	−6.1 (−10.1 to −2.2)	.003	417 (12.6)		397 (18.0)	−5.5 (−8.4 to −2.6)	<.001
Self-reported health was fair or poor at time of survey	1467 (66.5)	1024 (69.5)	−3.1 (−6.5 to 0.4)	.08	1236 (26.5)		868 (30.3)	−3.8 (−6.5 to −1.1)	.01

^a^ Data are for adults 65 years or older with low income (≤100% of the federal poverty level) who responded to the National Health Interview Survey from 2010 to 2013.

^b^ Those with chronic conditions had a limitation from any 1 of the following conditions: arthritis or rheumatism, heart problems, stroke problems, hypertension or high blood pressure, diabetes, lung or breathing problems, or cancer.

^c^ Data are presented as unweighted number (weighted percentage) of respondents unless otherwise indicated. Unweighted sample size was reported from 1 imputation wave. Weighted percentages and means were estimated using multiple imputation of 5 different samples that were defined based on 5 different imputed incomes.

^d^ Data are presented as percentage points for categorical data and counts for continuous data.

### Insurance Coverage

Table 3 shows results from difference-in-differences models of Medicaid coverage. Among older adults with low income and chronic condition limitations, expansion was associated with a differential increase in Medicaid enrollment (4.92 percentage points; 95% CI, 0.25-9.60 percentage points; *P* = .04) compared with nonexpansion. Among older adults without chronic condition limitations, there was no differential association between Medicaid expansion and Medicaid enrollment in expansion states (−0.24 percentage points; 95% CI, −3.06 to 2.57 percentage points; *P*  = .86).

**Table 3.** Changes in Medicaid Coverage, Health Care Use and Access, and Financial and Health Outcomes Associated With Medicaid Expansion^a^

Characteristic	Respondents with chronic condition limitations^b^		Respondents without chronic condition limitations	
	Coefficient estimate (95% CI)	No.	Coefficient estimate (95% CI)	No.
Had Medicaid coverage at time of survey	4.92 (0.25 to 9.60)	7144	−0.24 (−3.06 to 2.57)	14 675
Received medical care in person ≥10 times in past year	6.55 (1.16 to 11.93)	7132	1.96 (−1.25 to 5.18)	14 665
Had an office visit in past 2 wk	5.31 (0.10 to 10.51)	7140	1.80 (−1.07 to 4.66)	14 674
Office visits in past 2 wk, No.	0.12 (0.01 to 0.24)	7129	0.05 (−0.01 to 0.12)	14 656
Office visits in past 2 wk among those with a visit, No.	0.07 (−0.17 to 0.31)	2805	0.11 (−0.08 to 0.31)	3649
Had an overnight hospital stay in past year	−0.62 (−5.39 to 4.14)	7145	0.02 (−2.66 to 2.70)	14 692
Hospital stays in past year, No.	−0.13 (−0.54 to 0.29)	7130	0.01 (−0.05 to 0.07)	14 685
Hospital stays in past year among those with a stay, No.	−0.45 (−2.21 to 1.31)	2137	0.11 (−0.29 to 0.52)	1949
Had to delay or forgo care owing to cost at least once in past year	0.34 (−2.58 to 3.25)	7148	0.79 (−1.57 to 3.14)	14 688
Had problems paying medical bills in past year	0.29 (−4.64 to 5.22)	6421	2.48 (−0.62 to 5.57)	13 056
Self-reported health was fair or poor at time of survey	−0.15 (−6.10 to 5.79)	7151	4.28 (0.92 to 7.64)	14 698

^a^ Data are for adults 65 years or older with low income (≤100% of the federal poverty level) who responded to the National Health Interview Survey from 2010 to 2017. Weighted coefficients were estimated using multiple imputation for 5 different samples that were defined based on 5 different imputed incomes. The table presents coefficient estimates for *EXPANSION* × *POST*. Unweighted sample size was reported from 1 imputation wave. All regressions were weighted and included controls for age,^2^ educational level (less than high school, high school degree or General Educational Development Test, or some college; the omitted category was college or more), sex, race and ethnicity (Hispanic; non-Hispanic Black, or non-Hispanic White; the omitted category was other race and ethnicity), and state and year fixed effects. The difference-in-differences estimates presented reflect absolute, not relative, associations.

^b^ Those with a limitation from a chronic condition had a limitation from any 1 of the following conditions: arthritis or rheumatism, heart problems, stroke problems, hypertension or high blood pressure, diabetes, lung or breathing problems, or cancer.

### Health Care Use

Table 3 also shows results from difference-in-differences models of health care use. Among adults with chronic condition limitations residing in expansion states, Medicaid expansion was associated with a differential increase in the likelihood of receiving health care in person 10 or more times of 6.55 percentage points (95% CI, 1.16-11.93 percentage points; *P* = .02), a 16% increase compared with the period before expansion. Compared with nonexpansion, Medicaid expansion was associated with a differential increase in the likelihood of having an office visit of 5.31 percentage points (95% CI, 0.10-10.51 percentage points; *P* = .046), or 14%, and an 18% increase in the number of office visits (0.12 visits; 95% CI, 0.01-0.24 visits; *P* = .04). Medicaid expansion was not associated with differential use of inpatient hospital care, as measured by the likelihood of having an overnight hospital stay (−0.62 percentage points; 95% CI, −5.39 to 4.14 percentage points; *P* = .79) or the number of overnight hospital stays (−0.13 stays; 95% CI, −0.54 to 0.29 stays; *P* = .55). Among adults without chronic condition limitations, Medicaid expansion was not associated with a differential likelihood of having an office visit (1.80 percentage points; 95% CI, −1.07 to 4.66 percentage points; *P* = .21), number of office visits (0.05 visits; 95% CI −0.01 to 0.12 visits; *P* = .10), or any of the other health care use measures.

### Health Care Access and Financial and Health Outcomes

Medicaid expansion was not associated with differential cost-related access to care or difficulty paying medical bills in the group with chronic condition limitations or the group without these limitations (Table 3). Among respondents without chronic condition limitations, Medicaid expansion was associated with a differential increase in the likelihood of fair or poor self-reported health of 4.28 percentage points (95% CI, 0.92-7.64 percentage points; *P* = .01) compared with nonexpansion.

**Figure 1.**
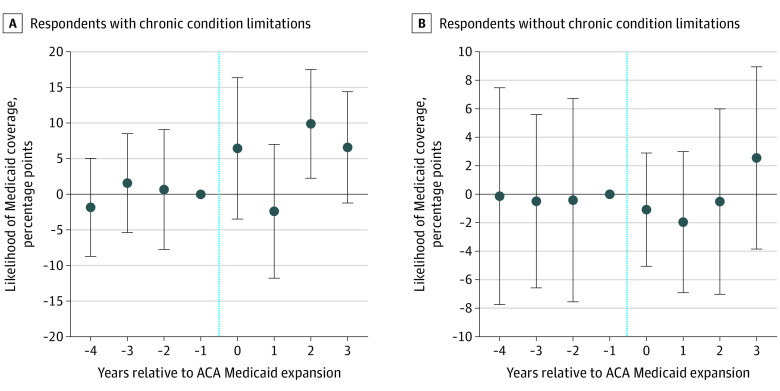
Event Study of Medicaid Coverage Among Older Adults With Low Income With and Without Chronic Condition Limitations Data are from respondents to the National Health Interview Survey from 2010 to 2017. Low income was defined as income at or below 100% of the federal poverty level. For *F* test of joint significance of coefficient estimates before Medicaid expansion, *P* = .73 (A) and *P* > .99 (B). Error bars represent 95% CIs, and the vertical blue lines indicate when the expansion was enacted. ACA indicates Patient Protection and Affordable Care Act.

**Figure 2.**
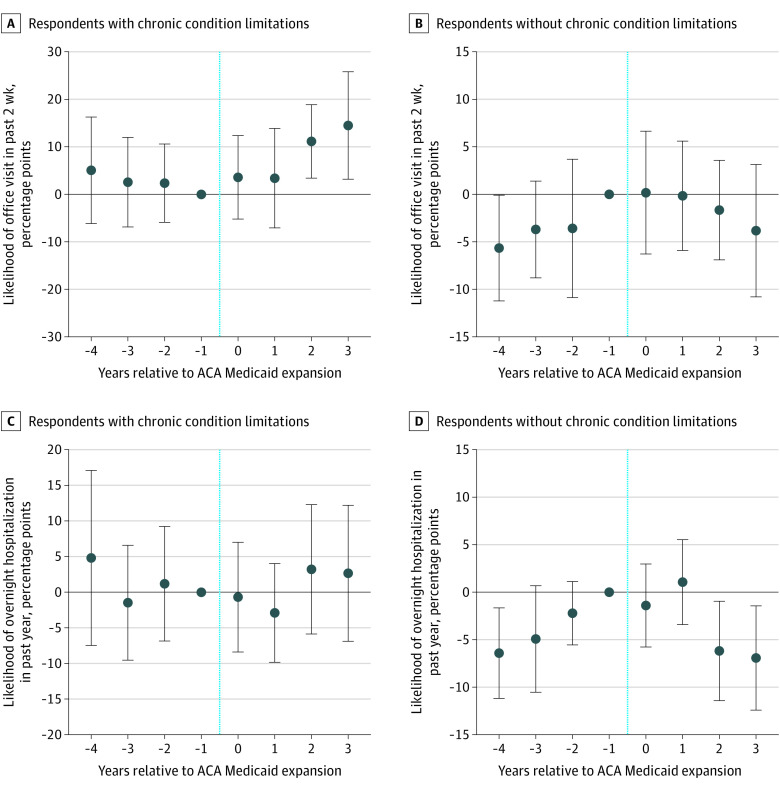
Event Study of Likelihood of an Office Visit in the Past 2 Weeks and Inpatient Hospitalization in the Past Year Among Older Adults With Low Income With and Without Chronic Condition Limitations Data are from respondents to the National Health Interview Survey from 2010 to 2017. Low income was defined as income at or below 100% of the federal poverty level. For *F* test of joint significance of coefficient estimates before Medicaid expansion, *P* = .83 (A), *P* = .25 (B), *P* = .64 (C), and *P* = .07 (D). Error bars represent 95% CIs, and the vertical blue lines indicate when the expansion was enacted. ACA indicates Patient Protection and Affordable Care Act.

Results from event study specifications showed that the parallel trends assumption was supported for all outcomes (Figure 1 and Figure 2 and eFigures 2-9 in the Supplement); associations observed in the difference-in-differences models tended to occur in the third year after Medicaid expansion, particularly for outpatient health care use. For example, the difference-in-differences estimate showed that over the entire postexpansion period, Medicaid expansion was associated with a differential 5.31 percentage point (95% CI, 0.10-10.51 percentage points; *P* = .046) increase in the likelihood of an office visit. In comparison, the event study results showed no statistically significant change in office visits in the year of and the year after expansion but showed increases of 11.13 percentage points (95% CI, 3.41-18.85 percentage points; *P* = .01) in year 3 and 14.48 percentage points (95% CI, 3.17-25.79 percentage points; *P* = .01) in year 4 (Figure 2A). Consistent with the difference-in-differences model results, event study analyses showed that for individuals with chronic condition limitations, Medicaid expansion was associated with an increase in Medicaid enrollment (Figure 1B), number of office visits (eFigure 3 in the Supplement), and likelihood of receiving care in person 10 or more times in the past year (eFigure 2 in the Supplement) but was not associated with hospital use (Figure 2C). Event study estimates showed no significant associations for those without chronic condition limitations (Figure 1B and Figure 2B and D).

eTable 1 in the Supplement shows the results of a difference-in-differences balance test examining whether inclusion of the sample of adults with chronic condition limitations was associated with Medicaid expansion. Results showed that Medicaid expansion was not associated with a differential likelihood of having a chronic condition limitation (1.90 percentage points; 95% CI −1.38 to 5.17 percentage points; *P* = .25) compared with nonexpansion.

In the sensitivity analyses of whether the results for respondents with chronic condition limitations were robust to the inclusion of additional covariates or imposition of different sample restrictions related to income, Medicare participation, or expansion timing, the signs and magnitudes of the estimated associations between Medicaid expansion and study outcomes were similar to our main results (eTables 2 and 3 in the Supplement). However, the associations were estimated less precisely in some cases.

Medicaid expansion was also associated with increases in 2 additional types of outpatient care among respondents with chronic condition limitations (eTable 4 in the Supplement). Conditional on having a telephone consultation or home health visit, Medicaid expansion was associated with a differential increase in the number of telephone consultations (1.01 consultations; 95% CI, 0.16-1.86 consultations; *P* = .02) and home health visits (1.48 visits; 95% CI, 0.30-2.66 visits; *P* = .02) compared with nonexpansion.

## Discussion

In this cross-sectional study, among older adults with low income and chronic condition limitations, Medicaid expansion was associated with increased Medicaid coverage and increased outpatient health care use but was not associated with inpatient health care use. We believe that these findings for health care use reflect demand-side responses to expanded coverage.

Dual Medicare and Medicaid enrollment may have increased because of the “welcome mat” effect, whereby individuals previously eligible for Medicaid enrolled after expansion because of increased program awareness, reduced enrollment costs, and lower stigma. Many studies have shown similar increases in Medicaid participation for other age groups eligible for Medicaid before expansion, and these mechanisms may also have contributed to the observed increase in Medicaid participation among older adults.^9,17,18,19,20,21,22,31^ For beneficiaries young enough to gain coverage under the 2014 expansions before age 65 years, firsthand experience with Medicaid may also have contributed to higher rates of dual Medicaid and Medicare enrollment at age 65 years.^23,32^

This increased Medicaid coverage may have been associated with substantial reductions in out-of-pocket expenditures. Even with Medicare, out-of-pocket costs can be burdensome, but for dual enrollees, Medicaid covers additional benefits and, of importance, covers Medicare cost-sharing to reduce the price of care to 0 for those with dual coverage. The literature shows that outpatient care is price sensitive for Medicare enrollees with low income and/or high health care needs.^33,34,35^

Policy changes may have had important supply-side responses that enhanced physician ability to accommodate increased demand. Evidence suggests that ACA Medicaid expansion was associated with increased physician acceptance of Medicaid patients^36,37^ and with increased geographic accessibility to physicians, especially primary care physicians.^38^ Furthermore, 19 states continued the ACA's Medicaid primary care fee bump past 2014.^39^ The ACA also increased the number of and support for Federally Qualified Health Centers,^40,41^ an important source of care for approximately 2.4 million Medicare beneficiaries.^42^

The only prior study, to our knowledge, on Medicaid expansion and individuals with dual enrollment in Medicare and Medicaid found no change in primary care use.^26^ Although our measure of outpatient care included both primary care physicians and specialists, this difference suggests the importance of distinguishing between persons with and without chronic condition limitations. When we estimated models using the pooled sample of all older adults with low income, we similarly found that Medicaid expansion was not associated with changes in health care use. Another difference is that the sample in our study included beneficiaries enrolled in Medicare Advantage and those in traditional Medicare. In addition, our event study specifications suggest that associations occurred in the third year after expansion, whereas the prior study examined only the year of expansion and the following year.^26^

The lagged associations found in our study also contrast with evidence of immediate associations of health care use and access among working-age adults^2,43^; however, recent work suggests that increases in use and access may plateau in the third year after expansion.^44^ The initial uptick in care among individuals aged 19 to 64 years may reflect pent-up demand among previously uninsured persons, and increases in care for the previously underinsured may follow.

Although Medicaid expansion was associated with increased health care use among older adults with chronic condition limitations, Medicaid expansion was not associated with changes in delaying care owing to cost or having trouble paying medical bills in our study. However, our analysis of these outcomes was limited by relatively small sample sizes, which may contribute to imprecise estimates; of note, the 95% CIs included large reductions in the likelihood of forgone care. Furthermore, the access measures in the NHIS person file strictly measure cost-related barriers and not other components of access, such as geographic accessibility, which was shown to improve after expansion in expansion states.^38,45^

### Limitations

This study has several limitations. For 39% of individuals 65 years or older, income was not reported and we relied on imputed income values. Thus, some classification error may have persisted in characterizing eligible individuals despite use of multiple imputation methods. In addition, limitations from chronic conditions were self-reported. Furthermore, the number of older adults with low income in the NHIS sample was small (21 859; 7153 with a limitation from a chronic condition), which limited our power to detect significant associations. Although the pattern of our results suggests that the increased health care use associated with Medicaid expansion may have been primarily attributable to increased Medicaid enrollment, we were unable to test this directly given the limited sample size.

## Conclusions

Previous research^23^ indicated that Medicaid expansion was associated with an indirect benefit of increased Medicaid coverage among older Medicare beneficiaries with low income, but it was unclear whether these changes differed between older adults with low income with and without chronic condition limitations or whether health care use changed among older adults with low income. Using a difference-in-differences design, we found that increases in Medicaid coverage were concentrated among older adults with low income with a chronic condition limitation. We also found increases in outpatient health care use in this group. These results suggest that there were indirect benefits associated with Medicaid expansion among older adults with complex health needs and that accounting for only the direct or intended effects associated with Medicaid expansion does not capture all changes associated with these insurance expansions.

## Notes

Supplement.
eAppendix.
**eFigure 1.** Flow Diagram of Sample Construction **eFigure 2.** Event Study Coefficient Estimates of Likelihood Respondent Received Medical Care in Person 10+ Times in Past Year, Respondents With Chronic Condition Limitations **eFigure 3.** Event Study Coefficient Estimates of Number of Office Visits in Past Two Weeks, Respondents With Chronic Condition Limitations **eFigure 4.** Event Study Coefficient Estimates of Number of Office Visits in Past Two Weeks Among Those With a Visit, Respondents With Chronic Condition Limitations **eFigure 5.** Event Study Coefficient Estimates of Number of Hospitalizations in Past Year, Respondents With Chronic Condition Limitations **eFigure 6.** Event Study Coefficient Estimates of Number of Hospitalizations in Past Year Among Those With a Stay, Respondents With Chronic Condition Limitations **eFigure 7.** Event Study Coefficient Estimates of Likelihood Respondent Had to Delay or Forego Care Owing to Cost in Past Year, Respondents With Chronic Condition Limitations **eFigure 8.** Event Study Coefficient Estimates of Likelihood Respondent Had Problems Paying Medical Bills in Past Year, Respondents With Chronic Condition Limitations **eFigure 9.** Event Study Coefficient Estimates of Likelihood Self-Reported Health Is Fair or Poor, Respondents With Chronic Condition Limitations **eTable 1.** Changes in Likelihood Respondent Has a Limitation From a Chronic Condition Among Older Adults With Low Income in States With Medicaid Expansion (Linear Probability Model), 2010-2017 NHIS **eTable 2.** Robustness to Inclusion of Potentially Endogenous Covariates and Different Definitions of the Included Sample; 2010-2017 NHIS **eTable 3.** Robustness to Exclusion of States That Expanded Medicaid Prior to 2014 or Adopted Expansion Mid-Year; NHIS 2010-2017 **eTable 4.** Changes in Additional Measures of Outpatient Utilization Among Older Adults With Low Income in States With Medicaid Expansion (Linear Probability Model and OLS), 2010-2017 NHIS
