# Supplementary material for: Association of Medicaid Expansion With Medicaid Enrollment and Health Care Use Among Older Adults With Low Income and Chronic Condition Limitations
Source: JAMA Health Forum. 2022 Jun 3;3(6):e221373. doi: 10.1001/jamahealthforum.2022.1373 (PMC9166222; doi:10.1001/jamahealthforum.2022.1373)
Supplement: Supplement. — eAppendix. eFigure 1. Flow Diagram of Sample Construction eFigure 2. Event Study Coefficient Estimates of Likelihood Respondent Received Medical Care in Person 10+ Times in Past Year, Respondents With Chronic Condition Limitations eFigure 3. Event Study Coefficient Estimates of Number of Office Visits in Past Two Weeks, Respondents With Chronic Condition Limitations eFigure 4. Event Study Coefficient Estimates of Number of Office Visits in Past Two Weeks Among Those With a Visit, Respondents With Chronic Condition Limitations eFigure 5. Event Study Coefficient Estimates of Number of Hospitalizations in Past Year, Respondents With Chronic Condition Limitations eFigure 6. Event Study Coefficient Estimates of Number of Hospitalizations in Past Year Among Those With a Stay, Respondents With Chronic Condition Limitations eFigure 7. Event Study Coefficient Estimates of Likelihood Respondent Had to Delay or Forego Care Owing to Cost in Past Year, Respondents With Chronic Condition Limitations eFigure 8. Event Study Coefficient Estimates of Likelihood Respondent Had Problems Paying Medical Bills in Past Year, Respondents With Chronic Condition Limitations eFigure 9. Event Study Coefficient Estimates of Likelihood Self-Reported Health Is Fair or Poor, Respondents With Chronic Condition Limitations eTable 1. Changes in Likelihood Respondent Has a Limitation From a Chronic Condition Among Older Adults With Low Income in States With Medicaid Expansion (Linear Probability Model), 2010-2017 NHIS eTable 2. Robustness to Inclusion of Potentially Endogenous Covariates and Different Definitions of the Included Sample; 2010-2017 NHIS eTable 3. Robustness to Exclusion of States That Expanded Medicaid Prior to 2014 or Adopted Expansion Mid-Year; NHIS 2010-2017 eTable 4. Changes in Additional Measures of Outpatient Utilization Among Older Adults With Low Income in States With Medicaid Expansion (Linear Probability Model and OLS), 2010-2017 NHIS [file jamahealthforum-e221373-s001.pdf]

Supplemental Online Content

McInerney M, McCormack G, Mellor JM, Sabik LM. Association of Medicaid expansion with Medicaid enrollment and health care use among older adults with low income and chronic condition limitations. *JAMA Health Forum.* 2022;3(6):e221373. doi:10.1001/jamahealthforum.2022.1373

eAppendix.

eFigure 1. Flow Diagram of Sample Construction

eFigure 2. Event Study Coefficient Estimates of Likelihood Respondent Received Medical Care in Person 10+ Times in Past Year, Respondents With Chronic Condition Limitations

eFigure 3. Event Study Coefficient Estimates of Number of Office Visits in Past Two Weeks, Respondents With Chronic Condition Limitations

eFigure 4. Event Study Coefficient Estimates of Number of Office Visits in Past Two Weeks Among Those With a Visit, Respondents With Chronic Condition Limitations

eFigure 5. Event Study Coefficient Estimates of Number of Hospitalizations in Past Year, Respondents With Chronic Condition Limitations

eFigure 6. Event Study Coefficient Estimates of Number of Hospitalizations in Past Year Among Those With a Stay, Respondents With Chronic Condition Limitations

eFigure 7. Event Study Coefficient Estimates of Likelihood Respondent Had to Delay or Forego Care Owing to Cost in Past Year, Respondents With Chronic Condition Limitations

eFigure 8. Event Study Coefficient Estimates of Likelihood Respondent Had Problems Paying Medical Bills in Past Year, Respondents With Chronic Condition Limitations

eFigure 9. Event Study Coefficient Estimates of Likelihood Self-Reported Health Is Fair or Poor, Respondents With Chronic Condition Limitations

eTable 1. Changes in Likelihood Respondent Has a Limitation From a Chronic Condition Among Older Adults With Low Income in States With Medicaid Expansion (Linear Probability Model), 2010-2017 NHIS

eTable 2. Robustness to Inclusion of Potentially Endogenous Covariates and Different Definitions of the Included Sample; 2010-2017 NHIS

eTable 3. Robustness to Exclusion of States That Expanded Medicaid Prior to 2014 or Adopted Expansion Mid-Year; NHIS 2010-2017

eTable 4. Changes in Additional Measures of Outpatient Utilization Among Older Adults With Low Income in States With Medicaid Expansion (Linear Probability Model and OLS), 2010-2017 NHIS

This supplemental material has been provided by the authors to give readers additional information about their work.

© 2022 McInerney M et al. *JAMA Health Forum.*

eAppendix

I. Software

All analyses were conducted using Stata\IC version 16.1 (StataCorp LP).

II. Flagging Chronic Conditions

We flagged individuals as having a limitation from a chronic condition if they report having a limitation arising from: arthritis or rheumatism; heart problems; stroke problem; hypertension or high blood pressure; diabetes; lung or breathing problems; or cancer. These correspond to the NHIS variables LAHCA3, LAHCA7, LAHCA8, LAHCA9, LAHCA10, LAHCA11, and LAHCA12 from the person file. These map to nine of the ten conditions used by the National Center for Health Statistics (NCHS) to identify the prevalence of multiple chronic conditions.[i] The only condition we were unable to include was hepatitis because it was not included in the list of follow up questions to the limitation question in the NHIS person file.

We chose to split the sample by whether the respondent experiences limitations from one or more chronic conditions and not whether the respondent was "ever told" they have a chronic condition as is frequently done in work using the NHIS because it permits us to compare the two groups. Whereas 32.7% of older adults with low income reported that they were currently experiencing at least one limitation from a chronic condition, the majority (78%) of older adults with low income have ever been told they have a chronic condition. In estimates not shown, for several outcomes the sample size for the complement of respondents who have never been told they have a chronic condition was too small to satisfy the minimum cell size for disclosure.

III. Identifying Individuals Likely Eligible for Medicaid

We restricted the NHIS to adults ages 65 and older who together with their spouse had income less than or equal to 100% federal poverty level (FPL). Adults ages 65 and older with income at or below 100% FPL and assets below $7,730 (single) and $11,600 (couple) are eligible for partial Medicaid through the Qualified Medicare Beneficiary (QMB) Medicare Savings Program, which pays Medicare premiums and cost sharing for Parts A and B. Our inclusion criteria also identified adults eligible for full Medicaid through the Aged, Blind, Disabled (ABD) pathway which also covers services that Medicare does not. In 2015, the ABD income eligibility limit ranged from 53% FPL for single adults in Connecticut to 100% FPL in 17 states; in most states, the ABD asset eligibility limit was $2000 for singles ($3000 for couples).[ii]

There are two main barriers to identifying these individuals in the NHIS data. First, in the NHIS only earned income is reported at the individual level while unearned income is reported at the family level. Since some seniors lived with family members aside from their spouse, we could not attribute all familial unearned income to the couple and instead must do an accounting exercise. Second, for 39% of the seniors in the years of our sample, exact income was not reported. Instead, NHIS provided five different imputed income values. We address our approach to these challenges below.

a. Accounting for family unearned income when computing total couple income

The NHIS reports total individual earned income (ERNYR) from the past year and total earned and unearned combined family income (FAMINCI). To calculate total earned and unearned spousal income combined, we followed the following procedure. For all individuals (i) in family (F), we summed the total individual earned income within a family to get gross family earned income. We then subtracted gross family earned income from total family income. The residual amount was assumed to be unearned income. We divided this amount among all individuals in the family who reported having some form of income. If there was a positive estimate of unearned income, but no individuals report having income, we divided the unearned income among all adults. If unearned income was estimated to be less than zero, we assumed there had been some error in reporting and set unearned

income equal to zero so that individuals were evaluated only based on their self-reported earned employment income. This reduced our sample relative to what we would have had if we incorporated negative unearned income. We summed the individual reported employment earnings with each individual's share of the family-level unearned income to get total individual income.

$$TotalIndividualIncome = EarnedIncome_i + \frac{FamilyIncome - \sum_{i \in family} EarnedIncome_i}{N}$$

If an individual is single, we used this total individual income as the evaluation income to compare to the federal poverty level. If individuals were married, the evaluation income was the sum of both spouses. In the 2010-17 NHIS, 46% of adults ages 65 and older resided in a family that had more than just the respondent and his or her spouse.

b. Multiple Imputation: Addressing Income Nonresponse

The other major challenge is the high rate of nonresponse to income questions among NHIS respondents. For the years 2010-2017, exact income was not reported for 39 percent of adults aged 65 and older. For these adults, the National Center for Health Statistics (NCHS) used imputation methods to generate five different imputed values for each variable.[iii] We followed NCHS guidelines to use these five different imputed values to conduct our analyses. In the exhibits, we reported the sample size for one of the five imputations. The sample size differs from imputation to imputation because respondents are only included in the sample when imputed income is at or below 100% FPL (i.e., they are excluded when imputed income is above 100% FPL). We report sample size from the first imputation. The multiple imputation procedure is outlined below.

We first used each different imputed value to separately calculate the evaluation income as defined in the previous section. For each of these five values, we then created a binary variable for being above or below the income threshold for sample inclusion. Each individual will then have five separate variables indicating sample inclusion which may not agree. We then used the multiple imputation package MI to run our analyses. We used the command MI SVYSET to register the complex structure of our data. For estimating sample means, we then used the command MI ESTIMATE, ESAMPVARYOK: SVY MEAN. To test the significance of differences in sample means, we used MI ESTIMATE TTESTTRANSFORM. To derive regression estimates, we used the MI ESTIMATE: REGRESS command along with NHIS survey sampling weights and estimated heteroskedasticity-robust standard errors clustered by state using the CLUSTER() option. The package runs a separate regression over each of the five imputation wave samples then combines point estimates and standard errors.

IV. Alternative Specifications, Sample Definitions, and Methods to Conduct Inference

In eFigure 1 we present a flow diagram of the sample construction.

In eFigures 2-9, we present results of an event study specification which allows us to both formally test the parallel trends assumption underlying the difference-in-differences analysis and understand how the effects evolve over time. In this model, we replace the term "POST x EXPANSION" in Equation (1) with a full set of indicator variables representing each year, relative to the expansion year, interacted with the EXPANSION indicator variable; non-expansion states are coded as zero in all years. We omit the indicator for the year prior to expansion in the state.

$$Y_{ist} = \sum_{t=-4, t \neq -1}^{3} \alpha_t EXPANSION_s * (Year = t)_t + \gamma_s + \gamma_t + X_i + \epsilon_{ist}$$

The Supplementary Figures (eFigures 2-9) plot the coefficients of these interaction terms and their confidence intervals and show the absence of differential pre-expansion trends between expansion and non-expansion state respondents for all outcomes. We formally tested this with an F-test of joint significance for the pre-period

coefficients. In every case, the p-value for this F-test exceeded 0.05. These event studies show that effects generally arose in the third year following expansion.

In eTable 1, we present the results of a balance test showing that the likelihood that a respondent reports a limitation from a chronic condition did not vary with ACA Medicaid expansion.

In eTable 2, we examine the robustness of the results to including potentially endogenous covariates and different definitions of the included sample. That is, we included controls for income, employment status, number of chronic conditions for which the respondent is experiencing limitations, number of limitations from activities of daily living (ADLs), family size, marital status, and the state unemployment rate. Estimates of the association of the Medicaid expansion with insurance coverage and utilization were robust to the inclusion of these variables.

Since Medicaid eligibility is determined after several adjustments are made to income, we first expanded the sample to include respondents with total income at or below 150% FPL, who were likely to be income-eligible for either QMB or full Medicaid. With the restricted-use NHIS data, we were able to examine effects for those who are income-eligible for full or ABD Medicaid in their state. Finally, it is possible that the welcome mat affected new enrollment in both Medicare and Medicaid, so we showed that our DD estimates are robust to including respondents not enrolled in Medicare. Our results are largely robust to these different sample definitions (eTable 2). The signs and magnitudes are similar to our baseline models, but the estimates on measures of office visits are less precise.

In eTable 3, we tested the robustness of the estimates to changes in the states included in the sample. In column (1), we exclude states that provided Medicaid coverage to childless adults with incomes up to 100% FPL or higher during the years 2010 through 2013 (i.e., District of Columbia, Delaware, Massachusetts, New York, and Vermont), as in Miller and Wherry (2017)[iv] and Ghosh, Simon, and Sommers (2019).[v] In column (2) we excluded states that had early (albeit small in some cases) Medicaid expansions (California, Connecticut, District of Columbia, Massachusetts, Minnesota, New Jersey, and Washington), as in Frean, Gruber, and Sommers (2017).[vi] In column (3), we excluded states that had mid-year expansions (Michigan, New Hampshire, Indiana, Louisiana, and Alaska), as in Courtemanche et al. (2017).[vii] The signs and magnitudes of the coefficient estimates are robust to these exclusions, though with a smaller sample size, the effect on Medicaid enrollment was estimated less precisely in the smallest sample and the effects on office visits were also estimated less precisely.

In eTable 4, we examined the effect of the Medicaid expansion on two other types of outpatient care: receiving advice or test results by phone in the past two weeks and home health care visits from health professionals in the past two weeks. Among respondents with a limitation from a chronic condition, there was no increase in the likelihood of receiving either type of care (-1.62, 95% CI -5.15 – 1.91, p=0.36) and (-0.33, 95% CI -5.78- 5.13, p=0.90), respectively. However, there was a statistically significant increase in the number of phone calls and number of home health visits, conditional on having any (1.01, 95% CI 0.16 – 1.86, p=0.02) and (1.48, 95% CI 0.30 – 2.66, p=0.02), respectively. There was no change in these types of outpatient care among respondents without a limitation from a chronic condition.

© 2022 McInerney M et al. *JAMA Health Forum.*

eFigure 1. Flow Diagram of Sample Construction

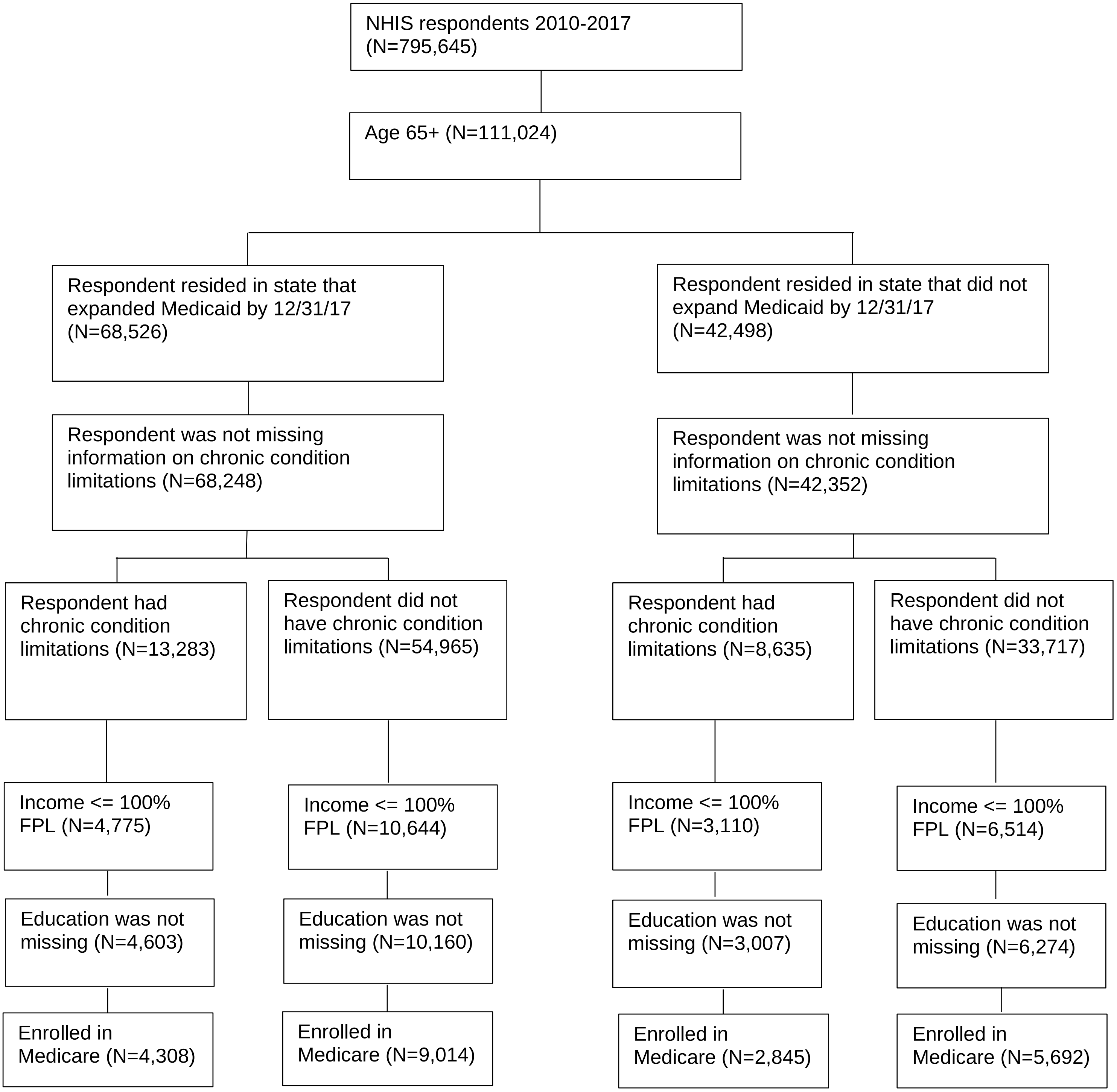

Notes: This flowchart depicts the sample size from one of the five imputations. Sample sizes reported in the text differ because some respondents are missing values of the dependent variable. Analysis sample (N=21,859) pools the bottom four boxes in the chart.

© 2022 McInerney M et al. *JAMA Health Forum.*

eFigure 2. Event Study Coefficient Estimates of Likelihood Respondent Received Medical Care in Person 10+ Times in Past Year, Respondents with Chronic Condition Limitations

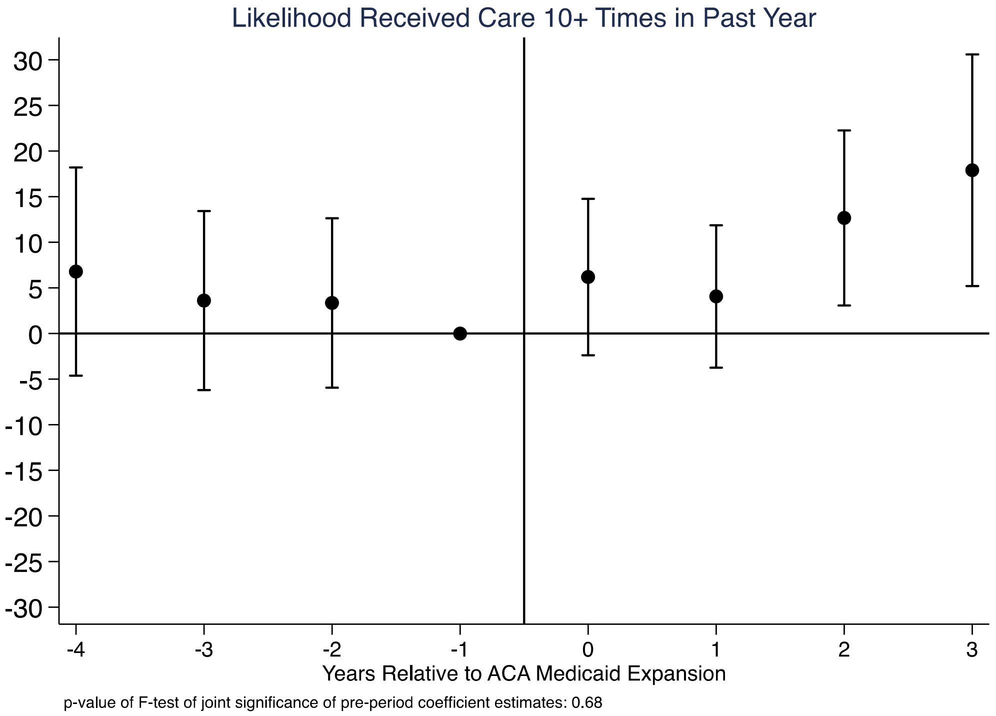

© 2022 McInerney M et al. *JAMA Health Forum.*

eFigure 3. Event Study Coefficient Estimates of Number of Office Visits in Past Two Weeks, Respondents with Chronic Condition Limitations

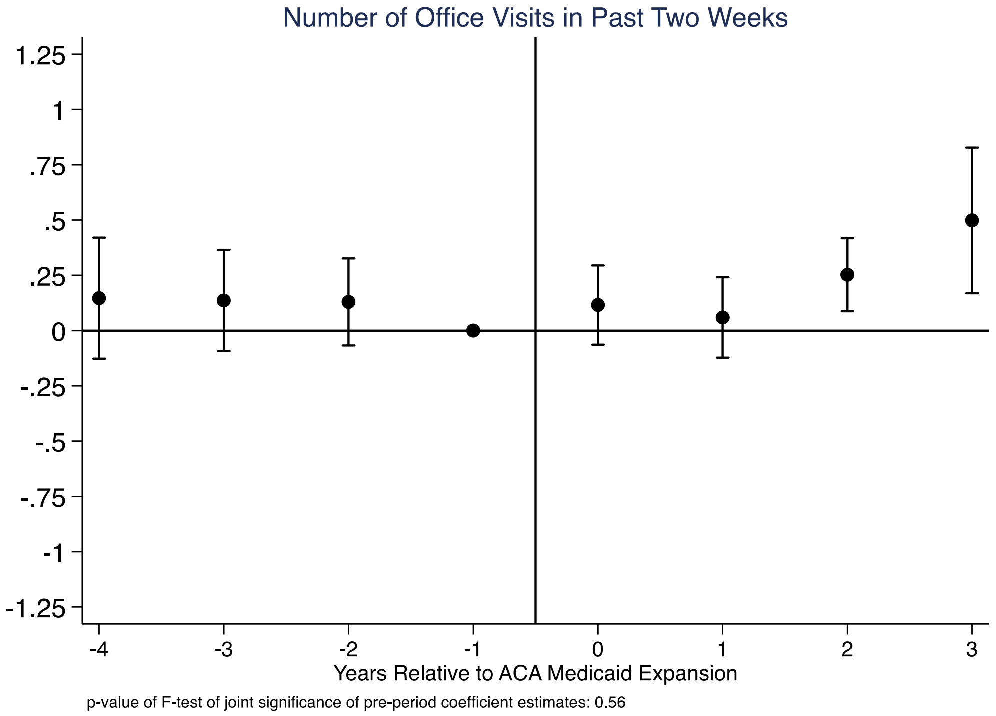

© 2022 McInerney M et al. *JAMA Health Forum.*

eFigure 4. Event Study Coefficient Estimates of Number of Office Visits in Past Two Weeks Among Those with a Visit, Respondents with Chronic Condition Limitations

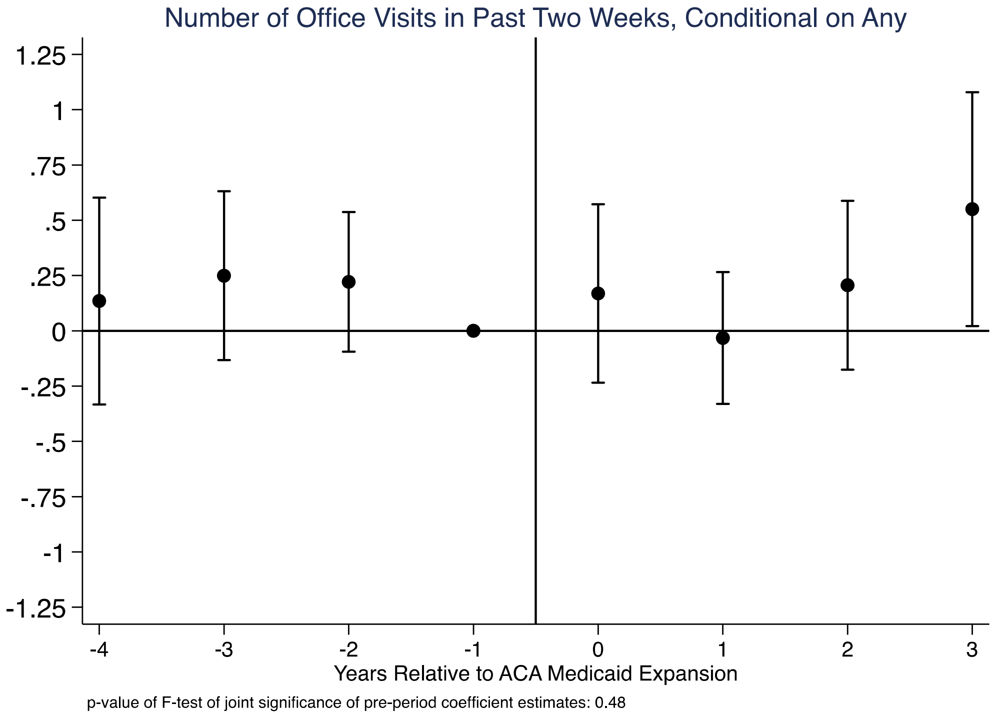

© 2022 McInerney M et al. *JAMA Health Forum.*

eFigure 5. Event Study Coefficient Estimates of Number of Hospitalizations in Past Year, Respondents with Chronic Condition Limitations

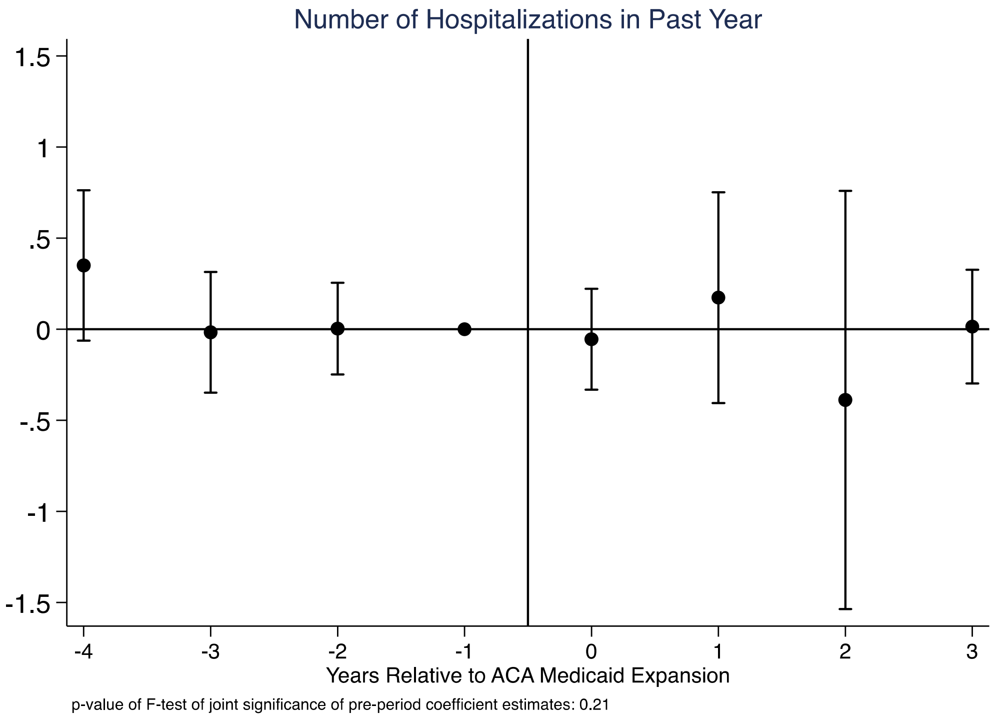

© 2022 McInerney M et al. *JAMA Health Forum.*

eFigure 6. Event Study Coefficient Estimates of Number of Hospitalizations in Past Year Among Those with a Stay, Respondents with Chronic Condition Limitations

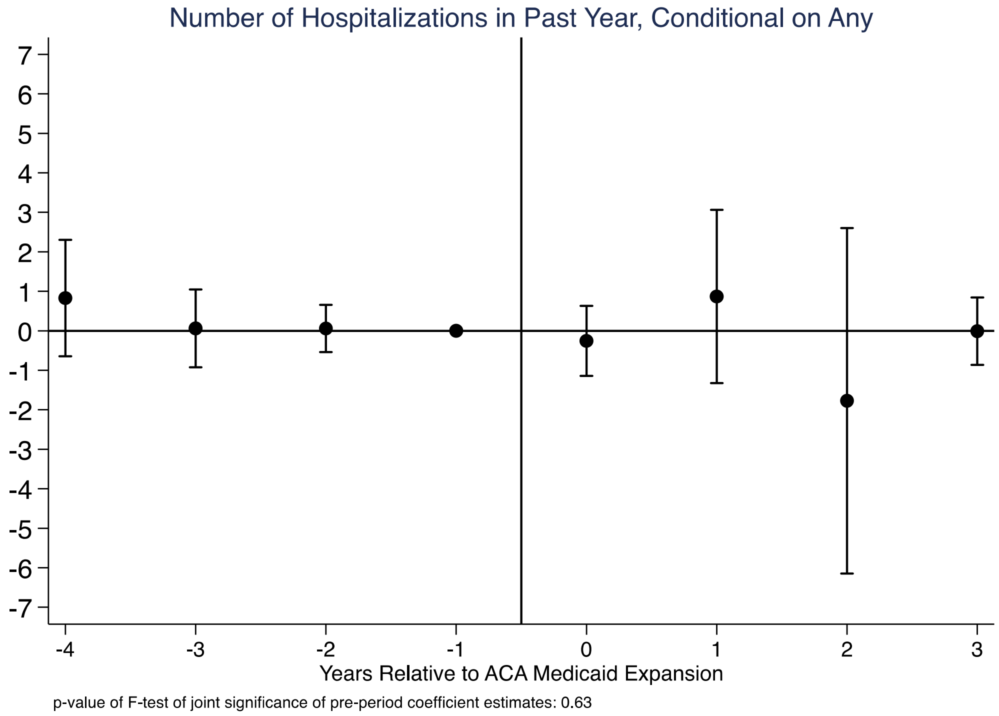

© 2022 McInerney M et al. *JAMA Health Forum.*

eFigure 7. Event Study Coefficient Estimates of Likelihood Respondent Had to Delay or Forego Care Owing to Cost in Past Year, Respondents with Chronic Condition Limitations

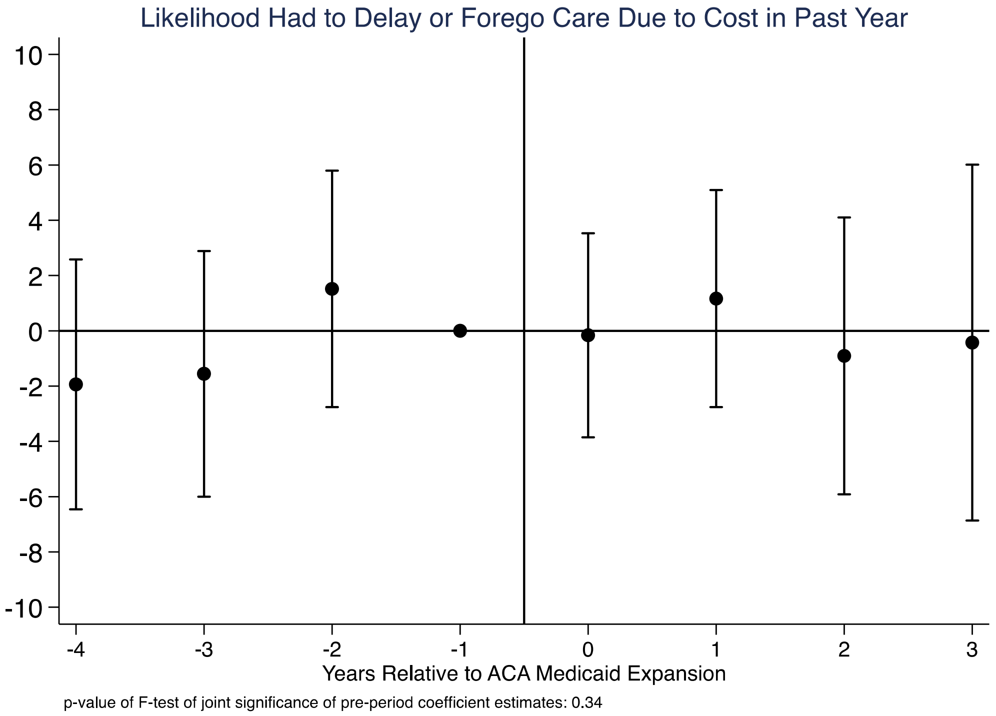

© 2022 McInerney M et al. *JAMA Health Forum.*

eFigure 8. Event Study Coefficient Estimates of Likelihood Respondent Had Problems Paying Medical Bills in Past Year, Respondents with Chronic Condition Limitations

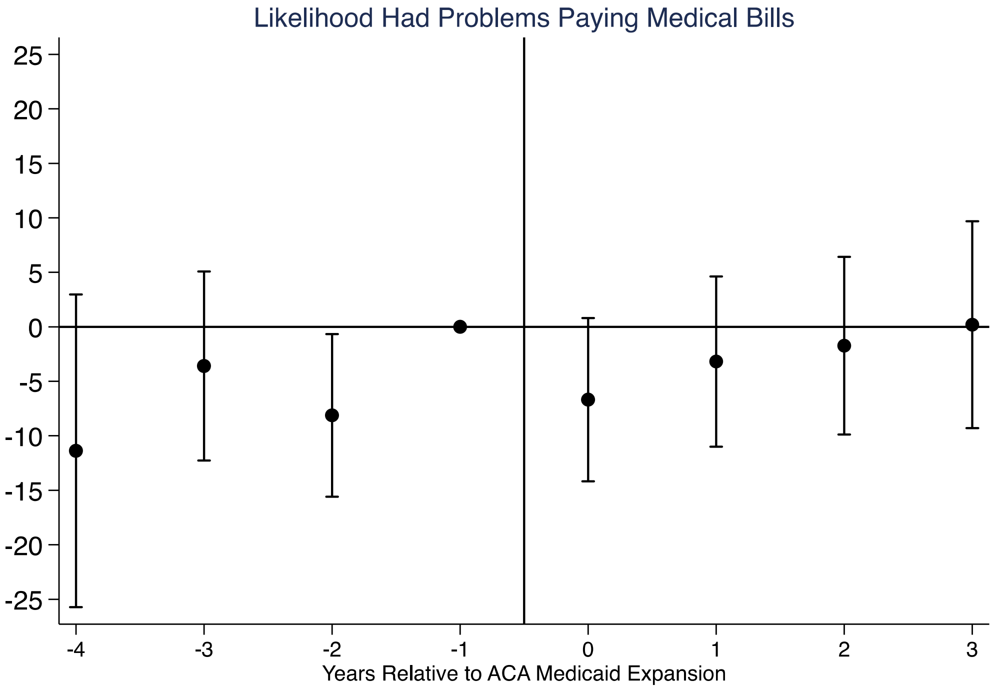

p-value of F-test of joint significance of pre-period coefficient estimates: 0.13

© 2022 McInerney M et al. *JAMA Health Forum.*

eFigure 9. Event Study Coefficient Estimates of Likelihood Self-Reported Health is Fair or Poor, Respondents with Chronic Condition Limitations

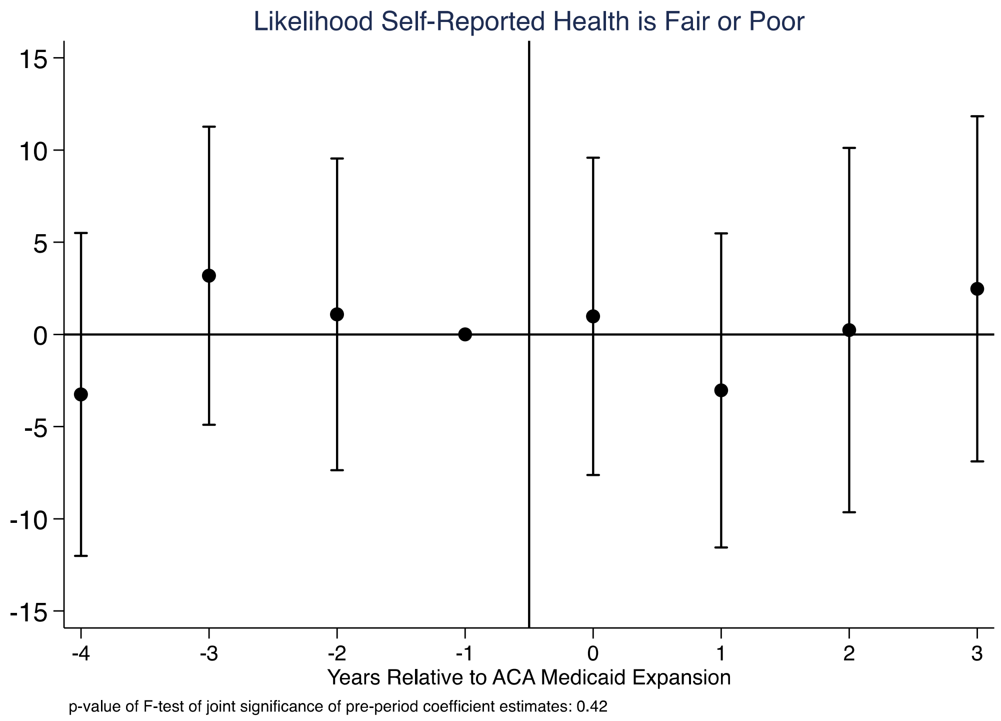

© 2022 McInerney M et al. *JAMA Health Forum.*

eTable 1. Changes in Likelihood Respondent Has a Limitation from a Chronic Condition Among Older Adults with Low Income in States with Medicaid Expansion (Linear Probability Model), 2010-2017 NHIS

	**Coefficient Estimate (95% CI)**	**N**
Has a limitation from a chronic condition	1.90 (-1.38 –5.17)	21,859

Note: Coefficients were estimated using multiple imputation over five different samples that were defined based on five different imputed incomes. Table presents coefficient estimate for EXPANSION x POST. Unweighted sample size reported from one imputation wave. The sample consists of respondents to the 2010-17 NHIS person file who are ages 65 and older and have income at or below 100% FPL. Those with a limitation from a chronic condition have a limitation from any one of the following conditions: arthritis or rheumatism; heart problems; stroke problem; hypertension or high blood pressure; diabetes; lung or breathing problems; or cancer. All regressions are weighted and include controls for age, age^2, education, sex, race, and state and year fixed effects. The DD estimate presented reflects absolute, not relative, effects.

eTable 2. Robustness to Inclusion of Potentially Endogenous Covariates and Different Definitions of the Included Sample; 2010-2017 NHIS

**Covariates:**	**Baseline + Potentially endogenous covariates**	**Baseline**	**Baseline**	**Baseline**
**Income Definition:**	**Income <=100% FPL**	**Income <= 150% FPL**	**Income <= 2015 State ABD Medicaid Eligibility Limit**	**Income <=100% FPL**
**Include non-Medicare enrollees?**	**No**	**No**	**No**	**Yes**
	**Coefficient estimate (95% CI)**			
Has Medicaid coverage (time of survey)	5.15 (0.14 – 10.16)	4.34 (0.95 – 7.73)	6.45 (0.66 – 12.23)	5.02 (0.48 – 9.55)
Received medical care in person 10+ times in past year	7.16 (2.14 – 12.19)	4.89 (1.31 – 8.47)	7.60 (1.73 – 13.47)	6.71 (1.22 –12.21)
Had an office visit, past 2 weeks	5.42 (0.24 – 10.60)	2.79 (-1.98 – 7.55)	4.76 (-1.39 – 10.90)	6.35 (1.41 – 11.29)
Number office visits, past 2 weeks	0.13 (0.01 – 0.25)	0.09 (-0.03 – 0.22)	0.08 (-0.06 – 0.22)	0.14 (0.02 – 0.26)
Number office visits (if any), past 2 weeks	0.10 (-0.16 – 0.36)	0.11 (-0.13 – 0.34)	-0.01 (-0.32 – 0.29)	0.09 (-0.16 – 0.35)
Had an overnight hospital stay, past year	-0.71 (-5.73 – 4.32)	-1.75 (-5.42 – 1.92)	0.36 (-4.35 – 5.07)	-0.15 (-4.43 –4.12)
Number of hospital stays, past year	-0.16 (-0.59 – 0.28)	-0.03 (-0.33 – 0.28)	-0.12 (-0.60 – 0.35)	-0.10 (-0.49 – 0.29)
Number of hospital stays (if any), past year	-0.54 (-2.33 – 1.25)	0.07 (-1.14 – 1.28)	-0.48 (-2.48 – 1.52)	-0.39 (-2.10 – 1.32)
Had to delay or forego care due to cost at least once, past year	0.46 (-2.48 – 3.39)	-0.71 (-3.95 – 2.54)	0.13 (-2.59 – 2.85)	0.65 (-2.09 – 3.38)
Had problems paying medical bills, past year	-0.15 (-5.14 – 4.84)	1.14 (-1.88 – 4.16)	-0.21 (-5.49 –5.08)	1.00 (-4.09 – 6.10)
Self-reported health is fair or poor (time of survey)	0.23 (-5.28 – 5.73)	0.41 (-4.67 – 5.49)	-0.03 (-6.48 –6.43)	0.78 (-4.91 – 6.47)
N	7,128	10,743	6,198	7,582

Note: Coefficients were estimated using multiple imputation over five different samples that were defined based on five different imputed incomes. Table presents coefficient estimate for EXPANSION x POST. Sample size reported from one imputation wave for the linear probability model predicting Medicaid participation. The sample consists of respondents to the 2010-17 NHIS person file who are ages 65 and older; have income at or below 100% FPL; and have a limitation from at least one of the following conditions: arthritis or rheumatism; heart problems; stroke problem; hypertension or high blood pressure; diabetes; lung or breathing problems; or cancer. All regressions are weighted and include controls for age, age^2,

education, sex, race, and state and year fixed effects. Potentially endogenous covariates include income, employment status, chronic conditions, # ADL, family size, marital status, and state unemployment rate. The DD estimates presented reflect absolute, not relative, effects.

eTable 3. Robustness to Exclusion of States that Expanded Medicaid Prior to 2014 or Adopted Expansion Mid-Year; NHIS 2010-2017

	Exclude States with Substantial Medicaid Coverage for Childless Adults pre-ACA	Exclude States that Adopted ACA Medicaid Expansion Early	Exclude States that expanded mid-year
	Coefficient Estimate (95% Confidence Interval)		
Has Medicaid coverage (time of survey)	6.14 (1.49 – 10.79)	4.52 (-1.29 – 10.32)	5.63 (0.86 – 10.39)
Received medical care in person 10+ times in past year	6.49 (0.67 – 12.30)	8.14 (3.29 – 12.98)	5.89 (0.38 – 11.40)
Had an office visit, past 2 weeks	6.15 (0.74 – 11.56)	5.60 (-0.16 – 11.37)	4.95 (-0.31 – 10.21)
Number office visits, past 2 weeks	0.14 (0.01 – 0.26)	0.08 (-0.05 – 0.22)	0.13 (0.001 – 0.25)
Number office visits (if any), past 2 weeks	0.09 (-0.15 – 0.33)	-0.03 (-0.29 – 0.23)	0.10 (-0.16 – 0.35)
Had an overnight hospital stay, past year	0.09 (-4.87 – 5.06)	-1.65 (-6.83 – 3.52)	-0.02 (-4.99 – 4.95)
Number of hospital stays, past year	-0.16 (-0.57 – 0.24)	-0.25 (-0.67 – 0.18)	-0.14 (-0.58 – 0.30)
Number of hospital stays (if any), past year	-0.72 (-2.45 – 1.00)	-0.80 (-2.49 – 0.88)	-0.54 (-2.39 – 1.31)
Had to delay or forego care due to cost at least once, past year	-0.004 (-3.07 – 3.06)	1.54 (-2.03 – 5.11)	-0.08 (-3.13 – 2.96)
Had problems paying medical bills, past year	0.36 (-5.06 – 5.78)	0.19 (-4.49 – 4.87)	-0.42 (-5.39 – 4.54)
Self-reported health is fair or poor (time of survey)	-0.11 (-6.52 – 6.30)	1.06 (-5.22 – 7.34)	0.81 (-5.60 – 7.22)
N	6,506	5,751	6,633

Note: Coefficients were estimated using multiple imputation over five different samples that were defined based on five different imputed incomes. Table presents coefficient estimate for EXPANSION x POST. Sample size reported from one imputation wave for the linear probability model predicting Medicaid participation. The sample consists of respondents to the 2010-17 NHIS person file who are ages 65 and older; have income at or below 100% FPL; and have a limitation from at least one of the following chronic conditions: arthritis or rheumatism; heart problems; stroke problem; hypertension or high blood pressure; diabetes; lung or breathing problems; or cancer. All regressions are weighted and include controls for age, age^2, education, sex, race, and state and year fixed effects. Column (1) excludes respondents residing in the District of Columbia, Massachusetts, New York, and Vermont because these states had coverage for childless adults prior to 2014 (as in Miller and Wherry, 2017 and Ghosh et al., 2019). Column (2) excludes respondents in California, Connecticut, the District of Columbia, Massachusetts, Minnesota, New Jersey, and Washington (as in Frean et al., 2017). Column (3) excludes respondents in Michigan, New Hampshire, Indiana, Louisiana, and Alaska (as in Courtemanche et al., 2017). The DD estimates presented reflect absolute, not relative, effects.

eTable 4. Changes in Additional Measures of Outpatient Utilization Among Older Adults with Low Income in States with Medicaid Expansion (Linear Probability Model and OLS), 2010-2017 NHIS

	Respondents with Chronic Condition Limitations					Respondents without Chronic Condition Limitations		
	Coefficient (95% CI)		Mean	N		Coefficient (95% CI)	Mean	N
Received advice or test results by phone, past 2 weeks	-1.62 (-5.15 – 1.91)		13.6	7,127		0.60 (-1.51 – 2.72)	6.0	14,667
Number phone calls to health professional, past 2 weeks	0.08 (-0.05 – 0.20)		0.3	7,107		0.01 (-0.04 – 0.06)	0.1	14,642
Number phone calls (if any), past 2 weeks	1.01 (0.16 – 1.86)		2.3	825		0.06 (-1.03 – 1.14)	1.9	886
Had home visit from health professional, past 2 weeks	-0.33 (-5.78 – 5.13)		17.2	7,153		1.20 (-0.18 – 2.58)	4.2	14,700
Number home health visits, past 2 weeks	0.27 (-0.08 – 0.62)		0.8	7,146		0.04 (-0.10 – 0.17)	0.2	14,693
Number home health visits (if any), past 2 weeks	1.48 (0.30 – 2.66)		4.8	1,236		-0.75 (-3.01 – 1.51)	6.1	643

Note: Coefficients were estimated using multiple imputation over five different samples that were defined based on five different imputed incomes. Table presents coefficient estimate for EXPANSION x POST. Sample size reported from one imputation wave. The sample consists of respondents to the 2010-17 NHIS person file who are ages 65 and older and have income at or below 100% FPL. Those with a limitation from a chronic condition report a limitation from any one of the following conditions: arthritis or rheumatism; heart problems; stroke problem; hypertension or high blood pressure; diabetes; lung or breathing problems; or cancer. All regressions are weighted and include controls for age, age^2, education, sex, race, and state and year fixed effects. Pre-period mean in expansion states reported. The DD estimates presented reflect absolute, not relative, effects.

[i] Boersma P, Black LI, Ward BW. Prevalence of Multiple Chronic Conditions Among US Adults, 2018. Prev Chronic Dis. 2020 Sep 17;17:E106. doi: 10.5888/pcd17.200130. PMID: 32945769; PMCID: PMC7553211.
[ii] Cornelio N, McInerney M, Mellor J, Roberts E, Sabik L. Increasing Medicaid Asset Test for People Eligible for Medicare and Medicaid Will Help Vulnerable Seniors. *Health Aff (Millwood).* 2021;40(12).
[iii] National Center for Health Statistics. Multiple Imputation of Family Income and Personal Earnings in the National Health Interview Survey: Methods and Examples. 2016. Available at: http://www.cdc.gov/nchs/data/nhis/tecdoc15.pdf
[iv] Miller S, Wherry LR. Health and Access to Care during the First 2 Years of the ACA Medicaid Expansions. *N Engl J Med*. 2017;376(10):947-956. doi:10.1056/nejmsa1612890
[v] Ghosh A, Simon K, Sommers B. The Effect of Health Insurance on Prescription Drug Use Among Low-Income Adults:Evidence from Recent Medicaid Expansions. *J Health Econ*. Jan 2019;63:64-80. doi:10.1016/j.jhealeco.2018.11.002
[vi] Frean M, Gruber J, Sommers B. Premium subsidies, the mandate, and Medicaid expansion: Coverage effects of the Affordable Care Act. *J Health Econ*. May 2017;53:72-86. doi:10.1016/j.jhealeco.2017.02.004

[vii] Courtemanche, CH, Marton J, Ukert B, Yelowitz A, Zapata D. Early Impacts of the Affordable Care Act on health insurance coverage in Medicaid expansion and non-expansion states. *J Policy Anal Manag*. 2017; 178-210.
